# Tungsten Inert Gas Welding of 6061-T6 Aluminum Alloy Frame: Finite Element Simulation and Experiment

**DOI:** 10.3390/ma17051039

**Published:** 2024-02-23

**Authors:** Yang Hu, Weichi Pei, Hongchao Ji, Rongdi Yu, Shengqiang Liu

**Affiliations:** 1 College of Mechanical Engineering, North China University of Science and Technology, Tangshan 063210, China; hy13472037552@163.com (Y.H.); liusq@ncst.edu.cn (S.L.); 2 School of Materials Science and Engineering, Zhejiang University, Hangzhou 310030, China; 3 TangShan ChenYang Sports Equipment Co., Ltd., Tangshan 063014, China; leoyurongdi@126.com

**Keywords:** TIG welding, free path, 6061-T6, optimization, numerical simulation

## Abstract

In order to address the irregularity of the welding path in aluminum alloy frame joints, this study conducted a numerical simulation of free-path welding. It focuses on the application of the TIG (tungsten inert gas) welding process in aluminum alloy welding, specifically at the intersecting line nodes of welded bicycle frames. The welding simulation was performed on a 6061-T6 aluminum alloy frame. Using a custom heat source subroutine written in Fortran language and integrated into the ABAQUS environment, a detailed numerical simulation study was conducted. The distribution of key fields during the welding process, such as temperature, equivalent stress, and post-weld deformation, were carefully analyzed. Building upon this analysis, the thin-walled TIG welding process was optimized using the response surface method, resulting in the identification of the best welding parameters: a welding current of 240 A, a welding voltage of 20 V, and a welding speed of 11 mm/s. These optimal parameters were successfully implemented in actual welding production, yielding excellent welding results in terms of forming quality. Through experimentation, it was confirmed that the welded parts were completely formed under the optimized process parameters and met the required product standards. Consequently, this research provides valuable theoretical and technical guidance for aluminum alloy bicycle frame welding.

## 1. Introduction

Aluminum alloy is a new type of structural material with significant advantages over steel, including corrosion resistance, lightweight properties, ease of processing and welding, and recyclability. It finds widespread applications in construction, transportation, and various other fields [1,2]. The 6xxx series Al-Mg-Si alloy, known for its excellent comprehensive properties, exhibits a high level of strength and plasticity. Compared to other aluminum alloys, it demonstrates superior impact toughness and can withstand greater ultimate deformation. Hence, the 6061-T6 aluminum alloy was chosen as the material for the bicycle frame in this study. In production and processing, the 6061-T6 aluminum alloy frame is typically welded using TIG (tungsten inert gas) welding. Due to the thin tube wall thickness of 2~3 mm in aluminum alloy frames and the specific shape of the tube connections, welding defects such as weld leakage, weld penetration, cracks, porosity, and poor weld formation commonly occur during thin-walled welding. To ensure that the welding meets production requirements and results in the desired form, it is essential to optimize the welding process parameters. The conventional trial-and-error method, relying on welding experiments, consumes substantial time and effort in optimizing the welding process, leading to unnecessary economic losses. Therefore, the development of corresponding control technology based on numerical simulation becomes imperative. Numerical simulations enable the effective prediction of the distribution of the welding temperature field and welding stress field [3,4,5], facilitating the optimization of welding process parameters [6,7,8,9,10]. This optimization enhances welding quality, reduces production costs, and enhances production efficiency in the welding process.

For the numerical simulation of the welding process, Nisar et al. simulated the laser welding process of AA5083 aluminum alloy in a straight path through ABAQUS v2020. They conducted a transient thermal analysis of the AA5083 aluminum alloy to study the effects of laser power and welding speed on the peak temperature of samples with different thicknesses, the aspect ratio of weld width and depth, and the width of the heat-affected zone [11]. Using the ANSYS platform, Doshi et al. performed transient thermal simulation of the welding process by utilizing an ANSYS APDL subroutine. They conducted a transient thermal simulation of pulsed MIG welding of a 1 mm thick AA6061-T6 thin plate. The study mainly focused on comparing the applicability and effects of various moving-heat-source models [12]. Liu et al. used ANSYS v2018 software to simulate the multi-pass TIG welding process of an RAFM steel plate. They numerically simulated the temperature field and stress field of TIG welding, supported by experimental studies [13]. Wang et al. introduced a sequential combination of thermal–mechanical and mechanical simulation methods. They conducted thermal–mechanical and mechanical simulations of double-pulse MIG welding of a 6061-T6 aluminum alloy sheet. The deformation behavior and load-bearing capacity of the welded joints, obtained through numerical simulation, were in good agreement with the corresponding experimental results. This demonstrates the feasibility and superiority of the established sequential simulation method, combining heat and force [14]. Lu et al. utilized the metal inert gas (MIG) welding method to weld aluminum alloy sheets and numerically simulated the welding process. They analyzed the welding temperature field and residual stress field of the butt joint. The model’s reliability was verified by comparing experimental data with simulation data [15]. Given the difficulty in writing free-path welding heat source subroutines, previous studies on numerical simulations of aluminum alloy welding have primarily focused on regular welding paths, with limited investigations into special-shaped pipe welding [16,17,18,19,20]. This paper conducts the secondary development of an irregular-path welding heat source subroutine for aluminum alloy frames. The TIG welding process parameters were optimized through numerical simulation using the response surface method [21,22,23], aiming to enhance welding quality and improve production efficiency [24,25,26].

## 2. Experimental Process and Materials

### 2.1. Materials and Methods

**Figure 1:**
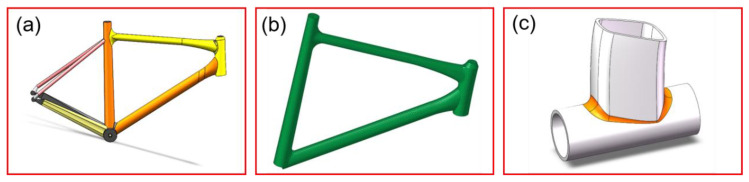
Frame’s intersecting line node parts and welding model. (**a**) Overall frame model. (**b**) Frame weldment model. (**c**) Weldment local model.

The material selected for this experiment is 6061-T6 aluminum alloy, and its chemical composition is presented in Table 1. The 6061-T6 sheet utilized in the experiment was purchased and its chemical composition was provided by the material supplier. In this study, the 3D modeling of the components is based on an actual bicycle frame. To facilitate specific analysis, a simple local modeling of its primary welding parts was conducted. The dimensions of the local modeling were as follows: a circular tube with a diameter of 44.7 mm, a length of 120 mm, and a thickness of 3.1 mm below, and an egg-shaped mouth-shaped tube with a curved radius, and a thickness of 2.85 mm above. The weld area is highlighted in Figure 1. The primary welding parameters of the TIG welding process are welding current, welding voltage, and welding speed. These three parameters are typically considered crucial for controlling welding quality and weldability characteristics. With the intersection line of the frame as the primary focus of the welding part, a welding analysis of the node position at the frame’s intersection line was conducted. The response surface method was employed to design corresponding experiments, optimizing the welding process parameters, with subsequent mechanical testing of welded parts and microstructure analysis. The fatigue performance of welded frame products was tested to validate the feasibility and accuracy of the welding optimization model.

### 2.2. Build Finite Element Model

The establishment of a welding finite element simulation model involves five main steps: model positioning, material setting, meshing, boundary condition definition, and loading of the heat source subroutine. The thermophysical performance parameters of 6061-T6 aluminum alloy material were obtained utilizing JmatPro v7.0 software and consulting the related literature [14]. The material’s relevant parameter settings are displayed in Table 2 and Table 3.

**Figure 2:**
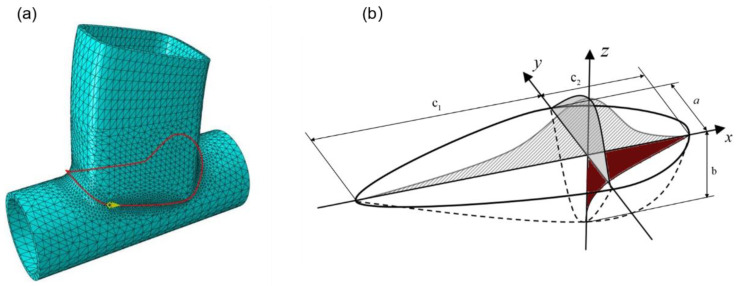
Welding path and pyrogen model. (**a**) Welding path diagram. (**b**) Double-ellipsoidal heat source model.

In order to carry out the welding numerical simulation more conveniently, the direct thermal–mechanical coupling method was used for the welding numerical simulation. Two physical field problems can be solved by using one element type, and the real coupling between heat and structure can be realized. Figure 2a is a schematic diagram of grid division and welding path. The red line is the welding trajectory, and the yellow arrow indicates the welding direction. The welding trajectory route is a specific free path of the intersecting line node. It is necessary to control the heat source movement trajectory of welding numerical simulation by writing specific subroutines. The heat source subroutine was written in Fortran language to control the welding path trajectory of the heat source movement in the welding numerical simulation process. According to the energy distribution characteristics of the heat source in the actual TIG welding of aluminum alloy, the heat source moves along the welding path, and the temperature of the heated position rises sharply and diffuses rapidly to the surrounding area. As the heat source moves, the heated position will form a tailing phenomenon of heat. The distribution on the surface of the welded plate is an asymmetric double-ellipsoid shape. A large temperature gradient will be formed around the weld, so the double-ellipsoid heat source model is used in this numerical simulation, as shown in Figure 2b [12]. The DFLUX subroutine interface provided by ABAQUS can realize the transformation simulation of moving the heat source load in time and space. At the same time, combined with the double-ellipsoid heat source distribution function, Fortran was used to complete the preparation of heat source load.

The double-ellipsoid heat source model takes into account the influence of the heat source movement on the heat flux distribution. The front of the heat source (the first half) is a 1/4 ellipsoid, and the rear (the second half) is another 1/4 ellipsoid. The heat flux density distribution function is expressed as [12]:

$$q(x,y,z,t)=\frac{6\sqrt{3}f_{f}}{abc_{1}\pi \sqrt{\pi }}\exp(-3(\frac{x^{2}}{a^{2}}+\frac{y^{2}}{b^{2}}+\frac{{\left(z-vt\right)}^{2}}{{c_{1}}^{2}}))$$

$$q(x,y,z,t)=\frac{6\sqrt{3}f_{r}}{abc_{2}\pi \sqrt{\pi }}\exp(-3(\frac{x^{2}}{a^{2}}+\frac{y^{2}}{b^{2}}+\frac{{\left(z-vt\right)}^{2}}{{c_{2}}^{2}}))$$

In the formula, $f_{f}$ and $f_{r}$ are heat flux distribution coefficients. $f_{f}+f_{r}=2$, $f_{f}$ takes 0.7, $f_{r}$ takes 1.3; a, b, $c_{1}$, and $c_{2}$ are the geometric dimensions of the molten pool [12]. Based on the above analysis, the heat source program was written in Fortran language and debugged to match the actual molten pool morphology. The DFLUX subroutine describes the model shape and moving state of the heat source. It has specific format requirements, and the overall format should conform to the characteristics of the Fortran statement. The basic way of writing its subprograms is as follows:
SUBROUTINE DFLUX(FLUX, SOL, KSTEP, KINC, TIME, NOEL, NPT, COORDS, JLTYP,    1    TEMP, PRESS, SNAME)   INCLUDE ‘ABA_PARAM.INC’   DIMENSION COORDS(3), FLUX(2), TIME(2)   real*8 Am, Bm, Cm, Dm, dmm, An, Bn, Cn, Dn, disn, Aw, Bw, Cw, Dw, dww   real*8 p1x, p1y, p1z, p2x, p2y, p2z, p3x, p3y, p3z   CHARACTER*80 SNAME      a = 3   b = 3   c = 3   c2 = 6   ratio = 0.5   ff = 0.66666667   fr = 1.33333333   CI = 280   U = 20   vel = 7   yita = 0.85   power = 1000*yita*U*CI   User coding to define FLUX(1) and FLUX(2)   RETURN   END

**Figure 3:**
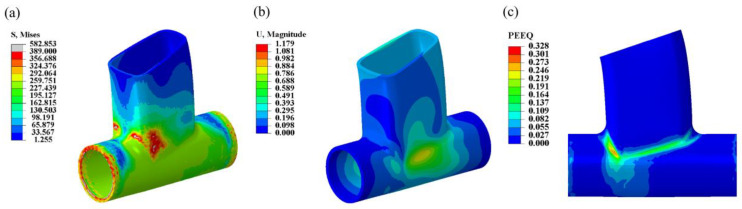
The welding numerical simulation results of the welding part of the frame intersecting line node. (**a**) Mises equivalent stress cloud diagram. (**b**) Displacement deformation cloud map. (**c**) Equivalent plastic strain cloud diagram.

Based on previous production experience, the welding process parameters were set as follows: the welding voltage was set to 20 V, the welding current was set to 280 A, and the welding speed was set to 7 mm/s. Subsequently, a welding numerical simulation was conducted using these parameters. In Figure 3, the results of the welding numerical simulation for the weldment are presented. Figure 3a displays the Mises equivalent stress distribution map of the weldment, with a maximum equivalent stress value of 582.853 MPa and a minimum equivalent stress value of 1.255 MPa. The region with the higher stress values is predominantly concentrated at the bending angle of the intersecting line. Figure 3b exhibits a cloud diagram depicting the post-weld displacement deformation of the weldment, where the maximum deformation value is recorded at 1.179 mm. Moreover, Figure 3c illustrates the cloud diagram showcasing the equivalent plastic strain distribution in the weldment post-welding, primarily observed in the weld area. The maximum value of equivalent plastic strain is calculated to be 0.328.

In summary, the weldment experiences excessive stress and strain post-welding due to the impact of welding process parameters. This directly affects the post-weld forming quality of the frame. Hence, it is imperative to optimize the welding process parameters in a rational manner. By obtaining the optimal welding process parameters, the forming quality of the weldment can be ensured.

## 3. Optimization of TIG Welding Process Parameters Based on RSM

### 3.1. Box–Behnken Experimental Design

The main factors influencing the welding quality in the TIG welding process are the welding current, the welding voltage, and the welding speed. To determine the optimal process parameters for welding, a three-factor, three-level, three-response Box–Behnken design test was established [27,28,29,30]. Table 4 presents the factor and level table of this experiment, while Table 5 displays the complete test table. The post-welding stress value, post-welding deformation, and welding temperature were selected as indicators. ABAQUS was employed to conduct the welding numerical simulations using the parameters listed in the table, and the results are summarized in the table.

### 3.2. Response Surface Model Fitting and Significance Analysis Test

**Figure 4:**
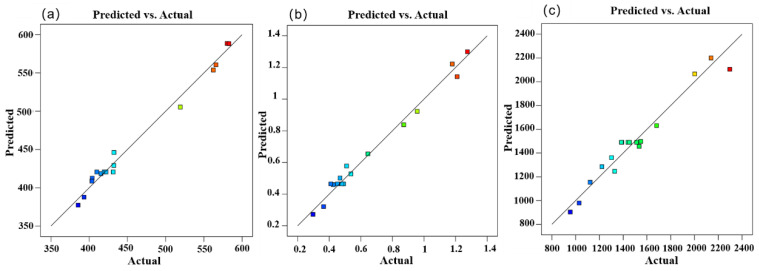
Correlation of prediction model (The colored squares represent the experimental group data). (**a**) Post-weld stress value model. (**b**) Post-weld deformation model. (**c**) Welding temperature model.

In Figure 4a–c, the predicted correlation models between post-welding stress value, post-welding deformation, and welding temperature are, respectively, illustrated. The results demonstrate a strong fitting effect of the model with minimal fitting errors. This indicates that the established response surface model provides accurate predictions for the fitting parameters. The coefficient of determination (R^2^) in the model reflects the goodness of fit, where a higher R^2^ value signifies a better correlation. The R^2^ values for the three models are 0.9876, 0.9855, and 0.9529, indicating a high degree of fit for all three models. The absolute differences between the predicted R^2^ and the adjusted R^2^ for the three models are all less than 0.2, indicating minimal errors in the response surface equations. Furthermore, the signal-to-noise ratios (22.7616, 23.0807, 19.9807) in the three models exceed 4, indicating sufficient signal strength and a strong discriminative ability of the model. This suggests that the model is suitable for optimizing the design of the welding process parameters. The variance analysis table and fitting statistics for the three models are provided below.

### 3.3. Analysis of Response Surface and Contour Map

**Figure 5:**
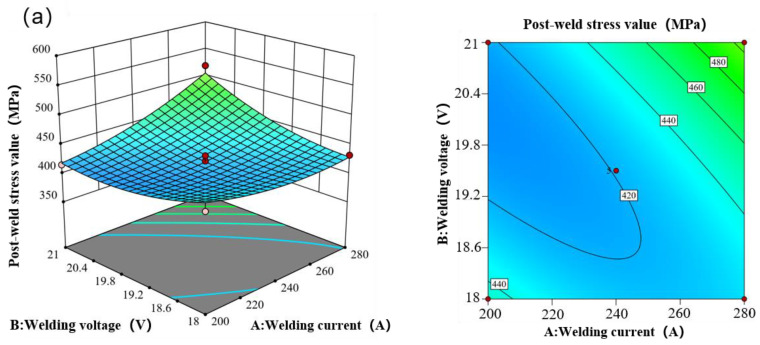

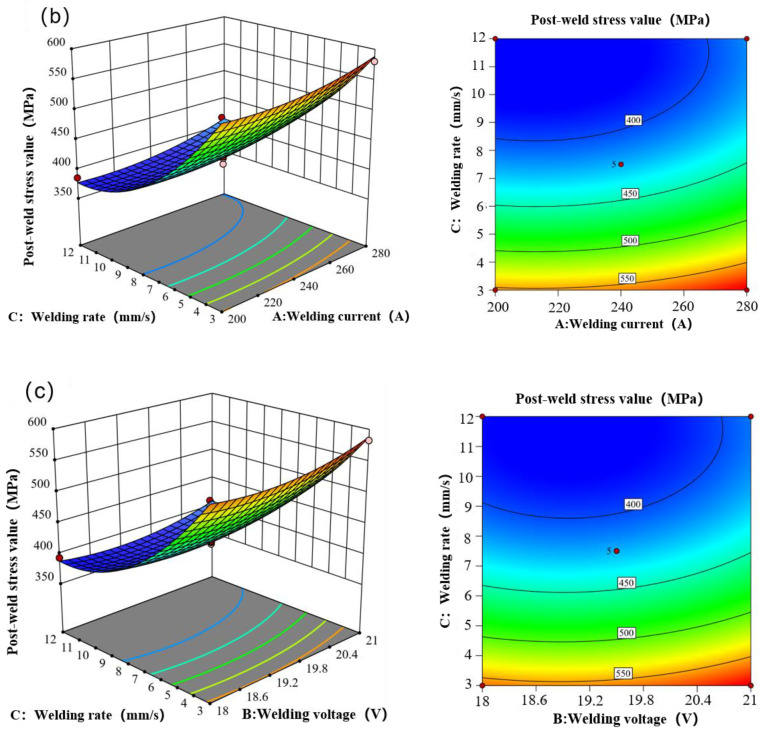
The response of the post-weld stress value of the weldment to the welding voltage, welding current, and welding rate. (**a**) The effect of the interaction between welding current and welding voltage on the stress value after welding. (**b**) The effect of the interaction between welding current and welding rate on the post-weld stress value. (**c**) The influence of the interaction between welding voltage and welding speed on the stress value after welding.

Figure 5 depicts the response surface of the post-welding stress value of the weldment concerning the variations in welding current, welding voltage, and welding speed. The evident curvature of the response surface signifies that the post-welding stress value of the weldment is notably impacted by the interaction of these three factors. A positive correlation exists between the post-welding stress value and both welding voltage and welding current—higher values of these parameters correspond to increased stress levels after welding. Conversely, there is a negative correlation between the post-welding stress value and welding speed, indicating that higher welding speed leads to reduced stress values post-welding. Table 6 presents the variance analysis table of the post-weld stress response surface model, while Table 7 displays the fitting statistical table of the post-weld stress response surface model. The dominance of welding speed in influencing the post-weld stress value is evident from the chart. The impact of the three forming process parameters varied in intensity; welding speed had the most significant influence, followed by welding voltage and welding current. Notably, interaction effects are observed among all three factors.

**Figure 6:**
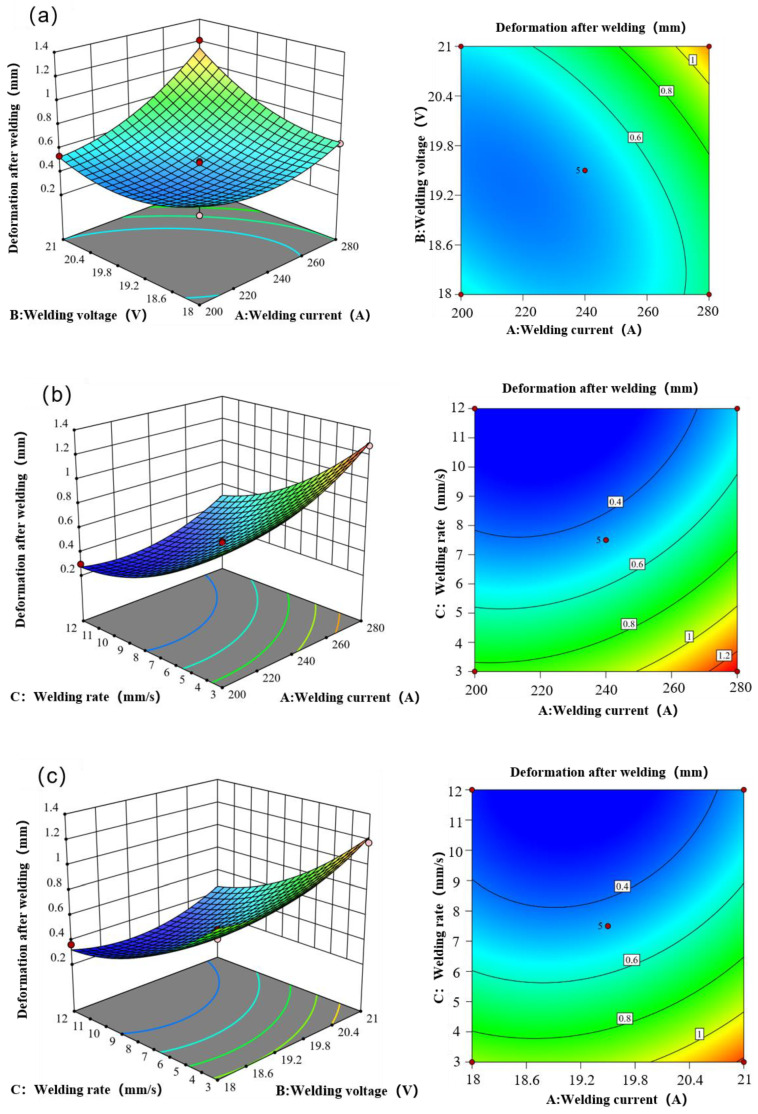
The response of the post-welding deformation of the weldment to the change of welding voltage, welding current, and welding rate. (**a**) The effect of the interaction between welding current and welding voltage on the deformation after welding. (**b**) The effect of the interaction between welding current and welding rate on the deformation after welding. (**c**) The influence of the interaction between welding voltage and welding speed on the deformation after welding.

Figure 6 illustrates the response surface of the post-weld deformation of the weldment in relation to changes in welding current, welding voltage, and welding speed. The post-weld deformation is significantly impacted by the interaction among these three factors. A positive correlation is observed between the post-weld deformation and both welding voltage and welding current—higher values of these parameters correspond to increased deformation post-welding. Conversely, a negative correlation is noted between the post-weld deformation and welding speed, indicating that higher welding speed results in reduced deformation post-welding. Table 8 provides the variance analysis table of the response surface model of post-weld deformation, while Table 9 presents the fitting statistical table of the response surface model of post-weld deformation. Analysis of the chart reveals that, in the post-welding deformation response surface model, the influence of the three factors varies in intensity, with welding speed having the most significant influence, followed by welding current and welding voltage. Additionally, interaction effects among all three factors are evident.

**Figure 7:**
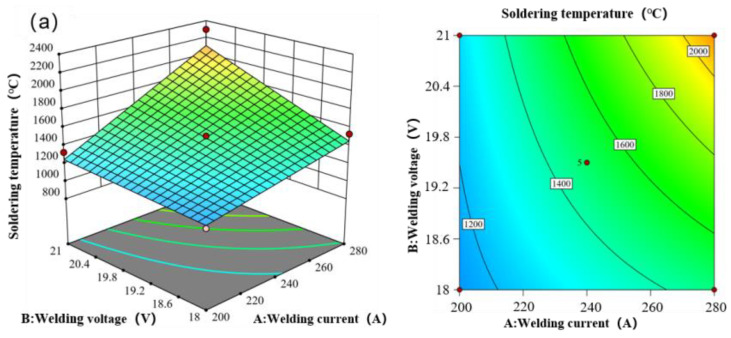

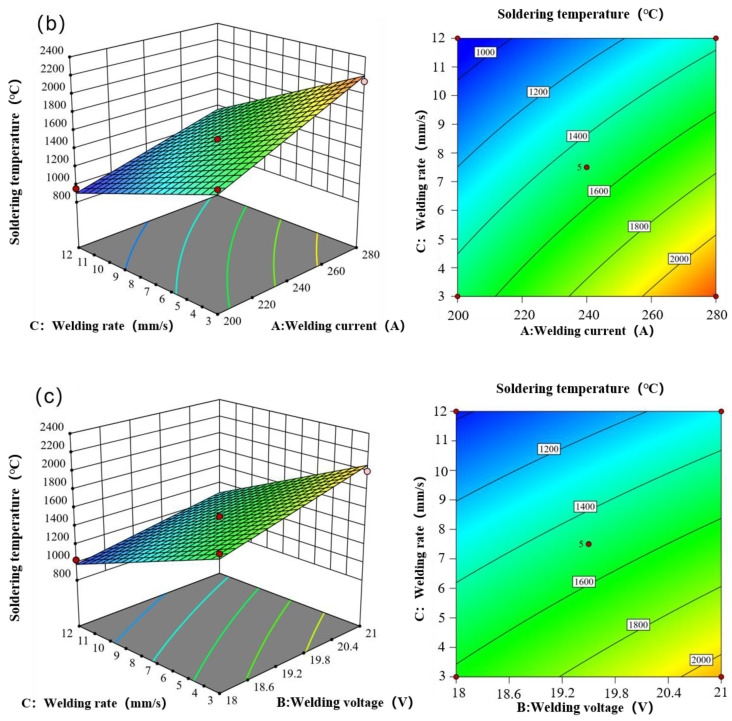
Response of welding temperature to welding voltage, welding current, and welding rate. (**a**) The influence of the interaction between welding current and welding voltage on welding temperature. (**b**) The influence of the interaction between welding current and welding rate on welding temperature is studied. (**c**) The influence of the interaction between welding voltage and welding rate on the welding temperature.

Figure 7 presents the response surface of the welding temperature of the weldment concerning changes in welding current, welding voltage, and welding speed. There exists a positive correlation between welding voltage, welding current, and welding temperature—higher values of welding voltage and welding current correspond to increased welding temperature. Conversely, a negative correlation is observed between welding speed and welding temperature, indicating that higher welding speed leads to lower welding temperature. Table 10 displays the variance analysis table of the welding temperature response surface model, while Table 11 showcases the fitting statistical table of the welding temperature response surface model. The chart indicates that the welding temperature is significantly influenced by the interaction of the three parameters.

The analysis above indicates that during the welding of the frame, the welding temperature, the post-weld stress value, and the post-weld deformation of the weldment are influenced by the interaction of the three process parameters: welding current, welding voltage, and welding speed. It is essential to consider the interaction between these process parameters thoughtfully to achieve the optimal welding process parameters. When aiming to minimize post-welding stress and post-welding deformation of the weldment, it is crucial for the simulated welding temperature value to align with the actual welding temperature. Based on these considerations, the response surface method was utilized to optimize the welding process parameters under these circumstances. Subsequently, the most appropriate welding process parameters were identified as following: a welding current of 241.145 A, a welding voltage of 19.5376 V, and a welding speed of 11.0634 mm/s. Considering practical adjustments, the optimized solution for this set of welding process parameters was fine-tuned to attain a welding current of 240 A, a welding voltage of 20 V, and a welding speed of 11 mm/s. Following this optimization, relevant welding experiments were conducted using this refined set of welding process parameters.

## 4. Result and Discussion

### 4.1. Numerical Simulation Results

#### 4.1.1. Welding Temperature Field Analysis

**Figure 8:**
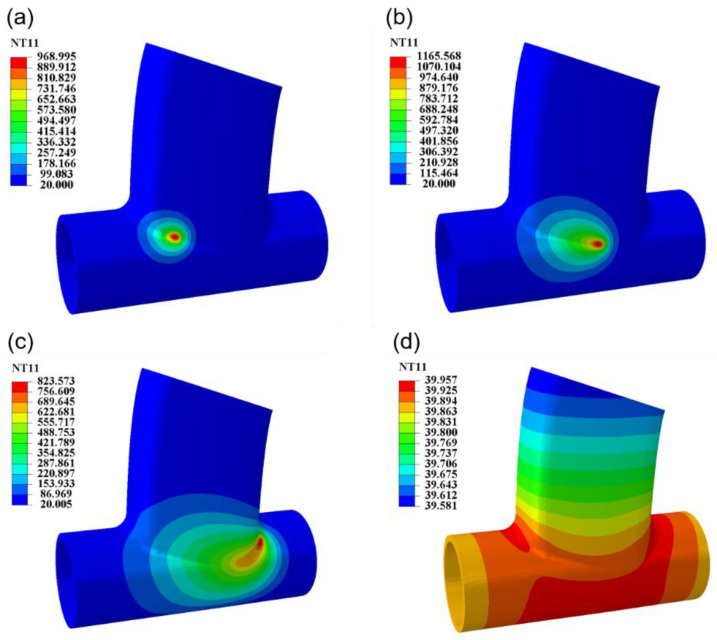
Welding temperature field model results. (**a**) Welding time of 1 s. (**b**) Welding time of 3 s. (**c**) Welding time of 6 s. (**d**) After 300 s cooling after welding.

A welding temperature field analysis was performed in order to optimize the welding process parameters and improve the forming quality of the weldment. According to the optimal process parameters obtained by the response surface, a welding numerical simulation was carried out. Figure 8 shows the dynamic process of temperature field change during welding. At the beginning of welding, the temperature of the heat source rises rapidly. As the welding progresses, a quasi-steady state is formed on the weldment; that is, the temperature of each point on the weld moves together with the heat source at a fixed value. As the welding ends, the heat source leaves the weld, and the weldment enters a cooling state. The isothermal line is gradually expanded until the temperature drops to room temperature. During welding, the heating rate of the weldment is much higher than the cooling rate. Therefore, the isotherms in the forward direction of the heat source are dense, and the rear isotherms are sparse. The heat source presents an asymmetric double-ellipsoid shape. The trailing phenomenon of heat is formed during the traveling of the heat source, and a large temperature gradient is formed around the weld [31]. From the simulation results, it can be seen that the change of the temperature field of the welding heat source can be well simulated by the heat source subroutine. It can reflect the correctness of the heat source program, and the heat source model can be used for the numerical calculation of the profile model.

**Figure 9:**
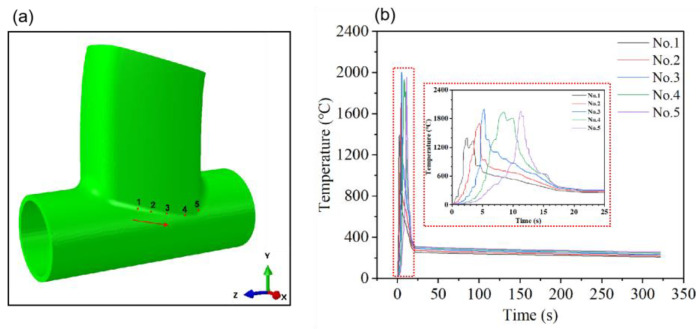
Welding temperature cycle curve before optimization. (**a**) The position of the weld is taken at 1–5 points. (**b**) Welding temperature cycle curve for the point taken.

**Figure 10:**
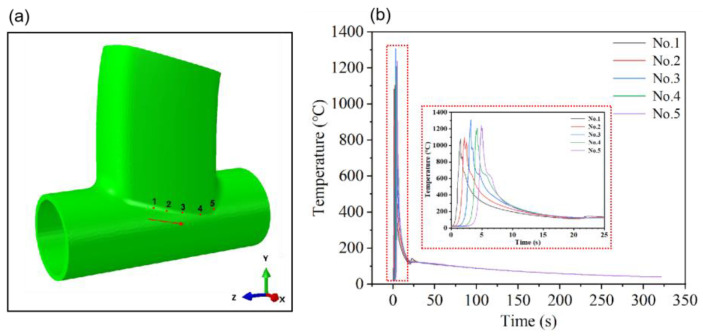
Optimized welding temperature cycle curve. (**a**) The position of the weld is taken at 1–5 points. (**b**) Welding temperature cycle curve for the point taken.

In this study, the joints at the intersecting line weld were selected as the research subjects, and the temperature variation patterns of each point on the weld were analyzed. The positions of the chosen points are illustrated in Figure 9a and Figure 10a. Figure 9b depicts the welding temperature cycle curve before optimization, whereas Figure 10b presents the optimized welding temperature cycle curve. Figure 9 reveals that the temperature range of the molten pool at nodes in the welding temperature cycle curve before optimization spans approximately 1200 to 2000 °C, with the highest temperature exceeding 2000 degrees Celsius. In a practical TIG welding of aluminum alloy thin-walled components, welding defects such as burn-through and cracking may occur. Conversely, Figure 10 displays the optimized welding temperature cycle curve, demonstrating a reduction in the molten pool temperature range of the welded joint to approximately 1000 to 1300 °C. The highest molten pool temperature is recorded at 1307 °C. A comparative analysis of Figure 9 and Figure 10 clearly indicates a significant reduction in the optimized welding temperature values, rendering them more compatible with the welding temperature requirements of the actual production process. This adjustment facilitates improved TIG welding of aluminum alloy thin-walled components [32].

#### 4.1.2. Welding Stress Field Analysis

**Figure 11:**
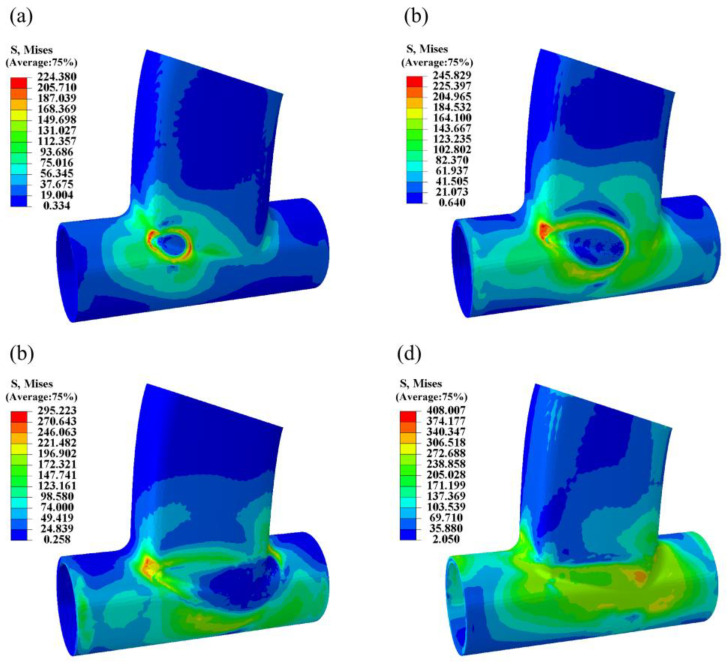
Welding stress field model results. (**a**) Welding time of 1 s. (**b**) Welding time of 3 s. (**c**) Welding time of 6 s. (**d**) After 300 s cooling post-welding.

**Figure 12:**
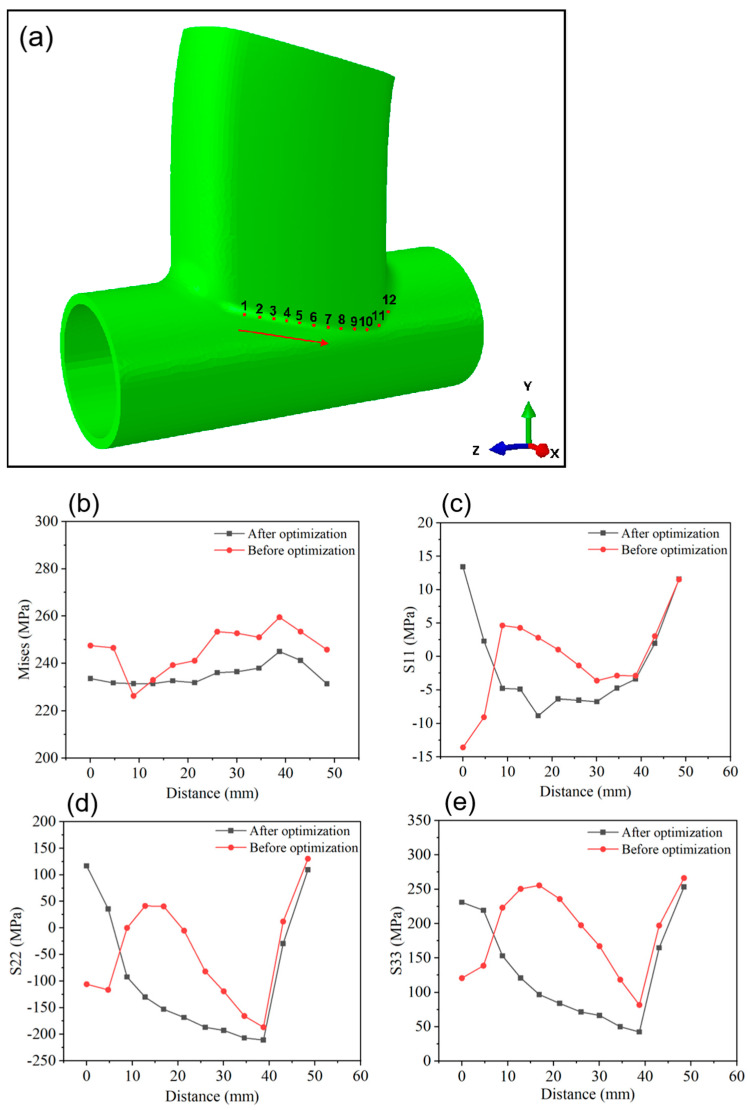
Comparison of welding stress field before and after optimization. (**a**) Point-taking diagram. (**b**) Mises stress value. (**c**) S11 stress value. (**d**) S22 stress value. (**e**) S33 stress value.

**Figure 13:**
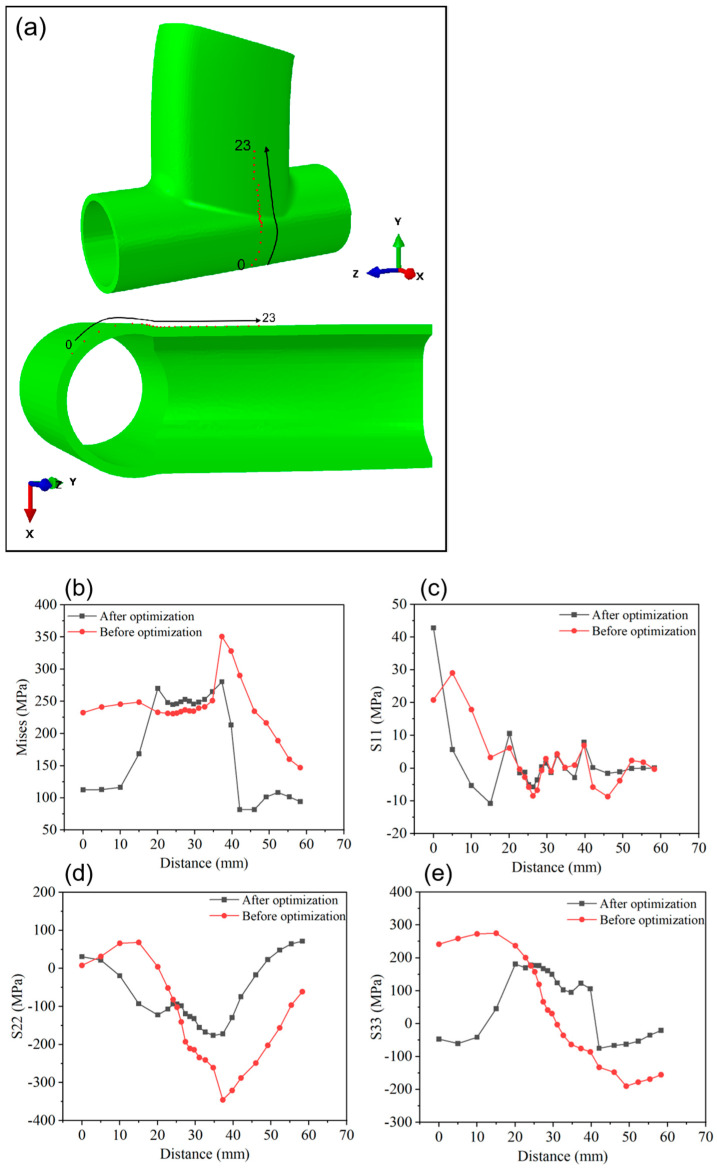
Comparison of welding stress field before and after optimization. (**a**) Point-taking diagram. (**b**) Mises stress value. (**c**) S11 stress value. (**d**) S22 stress value. (**e**) S33 stress value.

**Figure 14:**
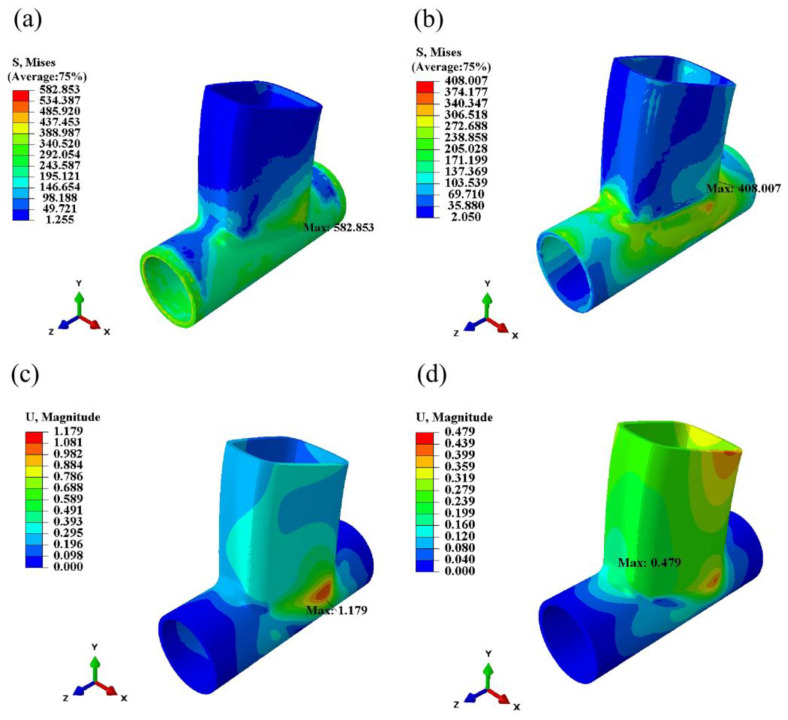
Comparison of stress and deformation before and after optimization. (**a**) Welding stress before optimization. (**b**) Optimized welding stress. (**c**) Welding deformation before optimization. (**d**) Optimized welding deformation.

The simulation results of the surface stress field are depicted in Figure 11, showcasing stress field contours at 1 s, 3 s, 6 s, and 300 s post-welding. Throughout the welding process, the localized melting of metal by the heat source forms a molten pool, where the stress value is nearly zero due to the flow and mixing of liquid metal. The rapid heating and cooling experienced by the metal in the weld area during welding result in thermal expansion and shrinkage, generating welding stress around the weld. As illustrated in Figure 11, the primary concentration of welding stress occurs around the weld, rather than inside the weld pool. Post-processing analysis of the simulation results for the weldment was conducted, with points selected as shown in Figure 12 and Figure 13. The Mises equivalent stress, transverse residual stress (S11), radial residual stress (S22), and longitudinal residual stress (S33) at these points along the weld path and perpendicular to it were compared and analyzed. Comparing Figure 12 and Figure 13, it is evident that, at the selected points, the equivalent stress value of the optimized weldment is lower than the equivalent stress value before optimization. Figure 14 presents a comparison of welding stress and welding deformation before and after optimization. The maximum welding stress value before optimization reached 582.853 MPa, whereas the maximum welding stress value after optimization was reduced to 408.007 MPa. Additionally, the maximum welding deformation before optimization was 1.179 mm, and after optimization, it decreased to 0.479 mm. This comparison clearly indicates that the optimized welding quality surpassed the quality observed before optimization.

### 4.2. Experiments and Analysis

#### 4.2.1. Structure Property Analysis

**Figure 15:**
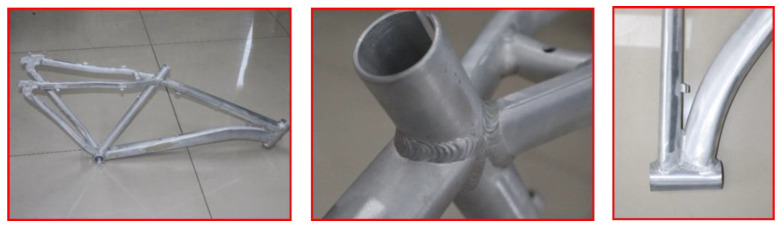
TIG welding experiment of 6061-T6 aluminum alloy bicycle frame.

According to the optimized welding process parameters (welding current of 240 A, welding voltage of 20 V, and welding speed of 11 mm/s), a TIG welding forming experiment was conducted on a 6061-T6 aluminum alloy bicycle frame. Prior to welding, the materials and parts were cleaned, ensuring the surfaces were free from oil, paint, and other contaminants, and the welding burrs were removed. The welding process specifications followed the Chinese national standard GB/T 19869.2-2012 [33], ‘Welding Procedure Qualification Test for Aluminum and Aluminum Alloys’. The experimental results are presented in Figure 15. No defects such as cracks, incomplete fusion, incomplete penetration, or burn-through were observed in the appearance inspection of the welded parts. The welding surface exhibited a well-spread appearance with dense fish-scale welds. The overall deformation of the weldment after welding was minimal, which closely matched the simulated post-weld deformation results. Overall, the welding quality of the entire frame was high, confirming the reliability of the welding numerical simulation and optimization design model.

**Figure 16:**
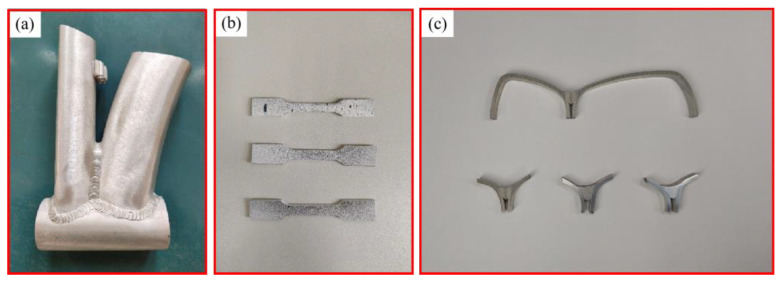
Sampling of welding test pieces. (**a**) Cutting sample of welding parts. (**b**) Tensile test specimen. (**c**) Metallographic specimen.

**Figure 17:**
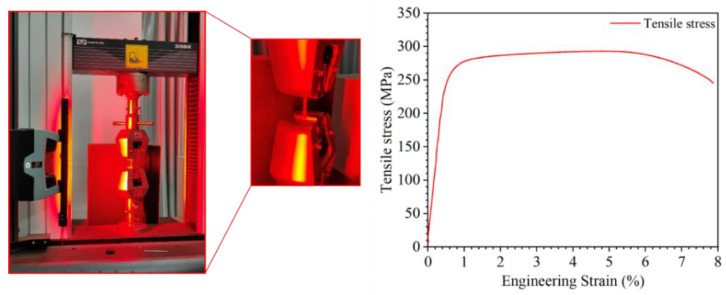
Tensile test.

The specimen was extracted by welding the experimental sample. As depicted in Figure 16, the welded components were sectioned using wire cutting to obtain samples for tensile testing and metallographic analysis. The mechanical properties and microstructural changes of the weldment were examined. The elongation and ultimate tensile strength (UTS) of all samples were assessed using a universal tensile testing machine (Instron, USA, model 3382). Testing was conducted at room temperature with a strain rate of 0.45 mm/min. The tensile specimens of the weldment were oriented vertically from the welding direction to ensure that the fusion zone fell within the specified length. The experimental findings are presented in Figure 17. The tensile strength of the frame amounted to 290 MPa, meeting the design criteria. The weld zone was sectioned and sampled, followed by the preparation of optical metallographic samples using standard mechanical polishing techniques. Subsequently, the samples underwent chemical etching with Keller reagent (HF:HCl:HNO_3_:H_2_O = 1:1.5:2.5:95) and were observed using an optical microscope (Leica Metallographic Microscope DMI8C) [34,35,36,37].

**Figure 18:**
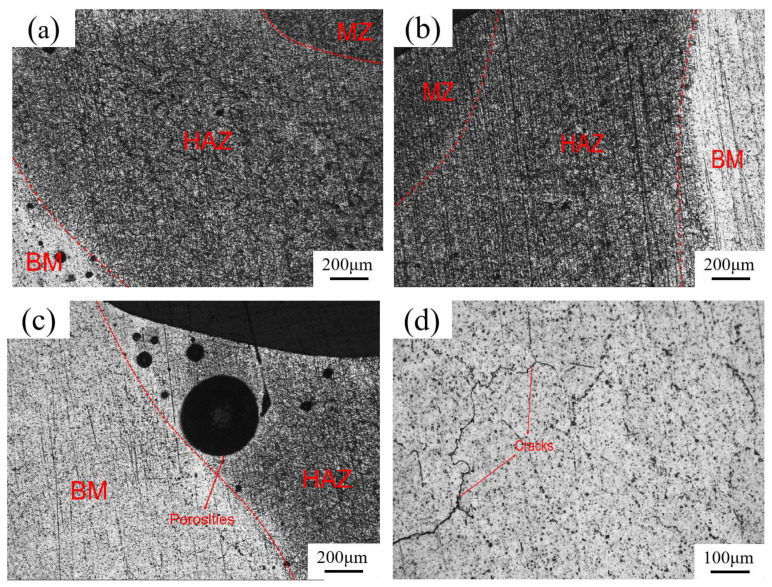
Metallographic structure of specified specimens in different locations. (**a**) A 50× microscopic view of the left side of the weld structure. (**b**) A 50× microscopic view of the right side of the weld structure. (**c**) There are certain pores at the base metal and the heat-affected zone. (**d**) There are certain microcracks in the base metal and the heat-affected zone.

Figure 18 illustrates the metallographic structure of the 6061-T6 aluminum alloy weld sample. After etching, the macrostructure of the weld can be categorized into three regions: the base metal, the heat-affected zone (HAZ), and the melting zone (MZ). These regions are symmetrically arranged along the weld center. The weld interfaces were identified as BM/HAZ and HAZ/MZ on the left side, and MZ/HAZ and HAZ/BM on the right side. Observation was conducted using a metallographic microscope [38,39].

Figure 18a–c depicts microscopic observations at 50× magnification. In these figures, the melting zone (MZ), the heat-affected zone (HAZ), and the base metal (BM) of the weld structure are clearly discernible. During the welding process, some internal pores often form in the composite interface area between the base metal and the heat-affected zone, as illustrated in Figure 18a,c. While the size of these pores varies, their number is not significant relative to the cross-section of the entire weld joint. Based on previous production experience, although some pores exist, they do not significantly impact the strength and reliability of welded joints. Hence, it can be inferred that the quality of the welded joint is relatively stable and meets general requirements. Figure 18d shows microscopic observation at 100× magnification of the welding relative composite interface section between the base metal and the heat-affected zone, revealing the presence of microcracks in this area.

The results of metallographic experiments are distinctive and provide valuable insights. Through such experiments, various defects in welded joints, including porosity, inclusions, and cracks, can be identified, allowing for a comprehensive evaluation of weldment quality. Additionally, the outcomes of metallographic experiments serve as a foundation for optimizing the welding process, facilitating enhancements in welding techniques and overall welding quality. Metallographic experiments conducted on weldments are crucial for evaluating welding quality, offering essential guidance for optimizing welding processes and ensuring product quality control.

#### 4.2.2. Frame Performance Testing

**Figure 19:**
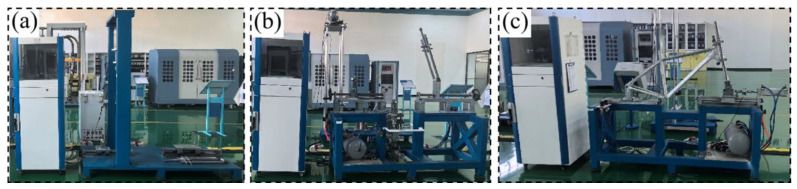
Frame performance test. (**a**) Vertical fatigue test of frame. (**b**) Foot pedal fatigue test of frame. (**c**) Horizontal repeated fatigue test of frame.

The bicycle frame manufactured using the optimal welding process parameters underwent performance testing to determine its adherence to standards. The frame was subjected to vertical fatigue testing, pedal fatigue testing, and horizontal repeated fatigue testing. The details of the performance tests are illustrated in Figure 19, with the corresponding results presented in Table 12. The data in Table 12 were obtained from the company’s testing organization, with all performance tests conducted in compliance with the Chinese national standard GB 3565.2-2022 [40]. Following the performance tests and inspections, the experimental outcomes were deemed satisfactory. This indicates that the bicycle frame produced using the best welding process parameters not only meets the performance test standards but also fulfills the practical requirements of production.

## 5. Conclusions

This paper primarily addresses the optimization design of TIG thin-wall welding process parameters for aluminum alloy bicycle frames and the practical application of optimized welding process parameters in production. Analysis of the macroscopic morphology and mechanical properties of the frame post-welding was conducted, along with related metallographic experiments on the weld. These investigations offer guidance for inexperienced practitioners, enhancing welding efficiency and weld quality, while also providing theoretical and technical guidance for welding aluminum alloy thin-walled special-shaped tubes for bicycles. The key conclusions are as follows:

With a focus on the TIG thin-walled welding of aluminum alloy bicycle frame, the welding numerical simulation was completed by compiling the free-path heat source subroutine in Fortran language based on an ABAQUS environment.

Using Box–Behnken experimental design and response surface method, the response surface model of post-welding stress value, post-welding deformation, and welding temperature evaluation index was established, and the influence of each process parameter on the evaluation index was determined. The optimum welding parameters were obtained: welding current of 240 A, welding voltage of 20 V, and welding speed of 11 mm/s.

Through welding test and performance test analysis, the optimization of TIG thin-walled welding forming process of 6061-T6 aluminum alloy bicycle frame was verified. The results show that the welding forming quality of the frame under the process parameters is high, meeting actual production needs, thus verifying the feasibility of the best welding process parameters.

## Figures and Tables

**Table 1:** Chemical composition of 6061-T6 aluminum alloy (wt.%).

Chemical Composition of 6061-T6 Aluminum Alloy				
Si	Fe	Cu	Mn	Mg
0.4~0.8	0.7	0.15~0.4	0.15	0.8~1.2
Cr	Zn	Ti	Al	Other
0.04~0.35	0.25	0.15	residuals	0.15

**Table 2:** Thermophysical properties of 6061-T6 aluminum alloy.

Alloy Material	Material Properties	20 °C	100 °C	200 °C	300 °C	400 °C	500 °C
6061-T6	Elastic modulus (MPa)	6.67 × 10^4^	6.08 × 10^4^	5.44 × 10^4^	4.31 × 10^4^	3.60 × 10^4^	3.00 × 10^4^
	Poisson ratio	0.334	0.339	0.344	0.349	0.356	0.363
	Thermal conductivity	119	121	126	130	138	145
	Specific heat (J kg^−1^ K^−1^)	900	921	1010	1050	1090	1130
	Yield stress (MPa)	250	225	190	133	20.8	8.6
	Plastic strain	0	0	0	0	0	0
	thermal expansion coefficient (mm/mm/°C)	2.23 × 10^−5^	2.28 × 10^−5^	2.47 × 10^−5^	2.55 × 10^−5^	2.67 × 10^−5^	2.70 × 10^−5^

**Table 3:** Welding model parameters.

Absolute zero (°C)	Boltzmann’s constant (W/mm^2^/°C)	Coefficient of convective heat transfer (mJ/mm^2^/s/°C)	Radiation heat transfer coefficient
−273.15	5.677 × 10^−11^	0.02	8.50 × 10^−4^
Density (t/mm^3^)	Latent heat (mJ/t)	Solidus temperature (°C)	Liquidus temperature (°C)
2.70 × 10^−9^	3.90 × 10^11^	615	655

**Table 4:** Experimental factor level.

Factor	Level		
	−1	0	1
Welding current (A)	200	240	280
Welding voltage (V)	18	19.5	21
Welding rate (mm/s)	3	7.5	12

**Table 5:** Box–Behnken analysis table.

Run	Factor 1 A: Welding Current (A)	Factor 2 B: Welding Voltage (V)	Factor 3 C: Welding Rate (mm/s)	Response 1 Stress (MPa)	Response 2 Deformation (mm)	Response 3 Temperature (°C)
1	280	19.5	12	403.949	0.468	1301.62
2	280	21	7.5	553.971	1.308	2296.86
3	240	19.5	7.5	387	0.4	1440
4	240	18	12	405.554	0.364	1029.57
5	240	19.5	7.5	420.514	0.524	1452.93
6	240	19.5	7.5	422	0.48	1513
7	240	18	3	565.88	0.957	1680.95
8	280	19.5	3	575.95	1.275	2139.58
9	240	19.5	7.5	419.5	0.491	1390
10	200	18	7.5	400.682	0.424	1121.81
11	280	18	7.5	431.733	0.645	1534.53
12	200	19.5	12	408.11	0.297	954.805
13	240	19.5	7.5	432	0.38	1385
14	200	21	7.5	415.422	0.537	1328.47
15	240	21	3	582.853	1.179	2001.55
16	200	19.5	3	562.451	0.872	1547.92
17	240	21	12	403.535	0.426	1221.12

**Table 6:** Analysis of variance of stress value after welding.

Source	Sum of Squares	df	Mean Square	F-Value	*p*-Value	
Model	81,495.28	9	9055.03	61.80	<0.0001	significant
A-current	2449.16	1	2449.16	16.71	0.0046	
B-voltage	1181.71	1	1181.71	80.6	0.0251	
C-welding speed	62,181.01	1	62,181.01	424.36	<0.0001	
AB	2702.86	1	2702.86	18.45	0.0036	
AC	0.0625	1	0.0625	0.0004	0.9841	
BC	11.35	1	11.35	0.0775	0.7888	
A2	700.98	1	700.98	4.78	0.0649	
B2	1107.72	1	1107.72	7.56	0.0285	
C2	10,312.40	1	10,312.40	70.38	<0.0001	
Residual	1025.70	7	146.53			
Lack of Fit	793.56	3	264.52	4.56	0.0885	not significant
Pure Error	232.14	4	58.04			
Cor Total	82,520.98	16				

**Table 7:** Fitting statistical scale of post-welding stress value.

Std. Dev.	Mean	C.V. %	R^2^	Adjusted R^2^	Predicted R^2^	Adeq Precision
12.10	457.68	2.64	0.9876	0.9716	0.8417	22.7616

**Table 8:** Variance analysis table of deformation after welding.

Source	Sum of Squares	df	Mean Square	F-Value	*p*-Value	
Model	1.60	9	0.1781	52.86	<0.0001	significant
A-current	0.2387	1	0.2387	70.86	<0.0001	
B-voltage	0.0959	1	0.0959	28.47	0.0011	
C-welding speed	0.9302	1	0.9302	276.11	<0.0001	
AB	0.0724	1	0.0724	21.84	0.0024	
AC	0.0135	1	0.0135	3.99	0.0858	
BC	0.0064	1	0.0064	1.90	0.2106	
A2	0.0697	1	0.0697	20.70	0.0026	
B2	0.0736	1	0.0736	21.84	0.0023	
C2	0.0764	1	0.0764	22.68	0.0021	
Residual	0.0236	7	0.0034			
Lack of Fit	0.0189	3	0.0063	5.33	0.0699	not significant
Pure Error	0.0047	4	0.0012			
Cor Total	1.63	16				

**Table 9:** Fitting statistical scale of post-welding deformation.

Std. Dev.	Mean	C.V. %	R^2^	Adjusted R^2^	Predicted R^2^	Adeq Precision
0.0580	0.6508	8.92	0.9855	0.9669	0.8099	23.0807

**Table 10:** Analysis of variance of welding temperature.

Source	Sum of Squares	df	Mean Square	F-Value	*p*-Value	
Model	2.068 × 10^6^	6	3.446 × 10^5^	33.75	<0.0001	significant
A-current	6.726 × 10^5^	1	6.726 × 10^5^	65.86	<0.0001	
B-voltage	2.742 × 10^5^	1	2.742 × 10^5^	26.85	0.0004	
C-welding speed	1.025 × 10^6^	1	1.025 × 10^6^	100.33	<0.0001	
AB	77,192.29	1	77,192.29	7.56	0.0205	
AC	14,987.27	1	14,987.27	1.47	0.2536	
BC	4163.48	1	4163.48	0.4077	0.5375	
Residual	1.021 × 10^5^	10	10,211.35			
Lack of Fit	91,165.01	6	15,194.17	5.55	0.0595	not significant
Pure Error	10,948.45	4	2737.11			
Cor Total	2.170 × 10^6^	16				

**Table 11:** Fitting statistical scale of welding temperature.

Std. Dev.	Mean	C.V. %	R^2^	Adjusted R^2^	Predicted R^2^	Adeq Precision
101.05	1490.57	6.78	0.9529	0.9247	0.8058	19.9807

**Table 12:** Performance test results.

Test Items	Standard Requires	Test Result
Vertical fatigue test of frame	A. Vertical downward force: 1200 N B. Test frequency: 2 Hz C. Test times: 100,000 times Judgment standard of test results: There shall be no visible cracks or fractures on the frame, no parts shall fall off, and the fiber frame shall be broken. The maximum deviation of the force from any direction in the middle position during the test shall not exceed 20% of the original value.	Tested for 100,000 times Pass
Foot pedal fatigue test of frame	A. Distance: Each pedal shaft is 150 mm away from the center of the frame. B. Load: 1200 N C. Force direction: The center of the frame is tilted 7.5 degrees outwards (accuracy within 0.5 degrees). D. Test times: 100,000 times Judgment standard of test results: There should be no visible cracks on the frame, and no parts of the shock absorber system should not fall off. For the carbon fiber frame, the maximum deviation of the force in any direction deviating from the middle position during the test shall not exceed 20% of the original value.	Tested for 100,000 times Pass
Horizontal repeated fatigue test of frame	A. Horizontal force F2: 1200 N; F3: 600 N (F2-The forward force; F3-The backward force) B. Test frequency: 2 Hz C. Test times: 100,000 times Judgment standard of test results: There must be no visible cracks or fracture on the frame, and no parts must fall off. The carbon fiber frame, the maximum deviation of the force generated by the force in any direction deviating from the middle position during the test shall not exceed 20% of the original value.	Tested for 100,000 times Pass

## Data Availability

Data are contained within the article.

## References

[1] Narsimhachary D., Bathe R.N., Padmanabham G., Basu A. (2014). Influence of temperature profile during laser welding of aluminum alloy 6061 T6 on microstructure and mechanical properties. Mater. Manuf. Process..

[2] Bansal A., Kumar M.S., Shekhar I., Chauhan S., Bhardwaj S. (2021). Effect of welding parameter on mechanical properties of TIG welded AA6061. Mater. Today Proc..

[3] Bansal A., Kumar M.S., Shekhar I., Chauhan S., Bhardwaj S. (2018). Numerical simulation and experimental validation of residual stress and welding distortion induced by laser-based welding processes of thin structural steel plates in butt joint configuration. Opt. Laser Technol..

[4] Pan M., Li Y., Sun S., Liao W., Xing Y., Tang W. (2022). A Study on Welding Characteristics, Mechanical Properties, and Penetration Depth of T-Joint Thin-Walled Parts for Different TIG Welding Currents: FE Simulation and Experimental Analysis. Metals.

[5] Costa S., Souza M.S., Braz-César M., Gonçalves J., Ribeiro J.E. (2021). Experimental and numerical study to minimize the residual stresses in welding of 6082-T6 aluminum alloy. AIMS Mater. Sci..

[6] Wu C., Wang C., Kim J.W. (2021). Bending deformation prediction in a welded square thin-walled aluminum alloy tube structure using an artificial neural network. Int. J. Adv. Manuf. Technol..

[7] Sathish T., Tharmalingam S., Mohanavel V., Ashraff Ali K.S., Karthick A., Ravichandran M., Rajkumar S. (2021). Weldability investigation and optimization of process variables for TIG-welded aluminium alloy (AA 8006). Adv. Mater. Sci. Eng..

[8] Trueba L., Torres M.A., Johannes L.B., Rybicki D. (2018). Process optimization in the self-reacting friction stir welding of aluminum 6061-T6. Int. J. Mater. Form..

[9] Naik A.B., Reddy A.C. (2018). Optimization of tensile strength in TIG welding using the Taguchi method and analysis of variance (ANOVA). Therm. Sci. Eng. Prog..

[10] Shao Q., Tan F., Li K., Yoshino T., Guo G. (2021). Multi-Objective Optimization of MIG Welding and Preheat Parameters for 6061-T6 Al Alloy T-Joints Using Artificial Neural Networks Based on FEM. Coatings.

[11] Nisar S., Noor A., Shah A., Siddiqui U., Khan S.Z. (2023). Optimization of process parameters for laser welding of A5083 aluminium alloy. Opt. Laser Technol..

[12] Doshi S., Jani D.B., Gohil A.V., Patel C.M. (2021). Investigations of heat source models in transient thermal simulation of pulsed MIG welding of AA6061-T6 thin sheet//IOP Conference Series: Materials Science and Engineering. IOP Conf. Ser. Mater. Sci. Eng..

[13] Liu S., Sun J., Wei F., Lu M. (2018). Numerical simulation and experimental research on temperature and stress fields in TIG welding for plate of RAFM steel. Fusion Eng. Des..

[14] Wang J., Chen X., Yang L., Zhang G. (2022). Sequentially combined thermo-mechanical and mechanical simulation of double-pulse MIG welding of 6061-T6 aluminum alloy sheets. J. Manuf. Process..

[15] Lu Y., Zhu S., Zhao Z., Chen T., Zeng J. (2020). Numerical simulation of residual stresses in aluminum alloy welded joints. J. Manuf. Process..

[16] Tlili I., Baleanu D., Mohammad Sajadi S., Ghaemi F., Fagiry M.A. (2022). Numerical and experimental analysis of temperature distribution and melt flow in fiber laser welding of Inconel 625. Int. J. Adv. Manuf. Technol..

[17] Iqbal M.P., Jain R., Pal S.K. (2019). Numerical and experimental study on friction stir welding of aluminum alloy pipe. J. Mater. Process. Technol..

[18] Duggirala A., Kalvettukaran P., Acherjee B., Mitra S. (2021). Numerical simulation of the temperature field, weld profile, and weld pool dynamics in laser welding of aluminium alloy. Optik.

[19] Chen L., Mi G., Zhang X., Wang C. (2021). Effects of sinusoidal oscillating laser beam on weld formation, melt flow and grain structure during aluminum alloys lap welding. J. Mater. Process. Technol..

[20] Kik T. (2020). Computational techniques in numerical simulations of arc and laser welding processes. Materials.

[21] Azevedo S.C., de Resende A.A. (2021). Effect of angle, distance between electrodes and TIG current on the weld bead geometry in TIG-MIG/MAG welding process. Int. J. Adv. Manuf. Technol..

[22] dos Santos Paes L.E., Andrade J.R., Lobato F.S., dos Santos Magalhães E., Ponomarov V., de Souza F.J., Vilarinho L.O. (2022). Sensitivity analysis and multi-objective optimization of tungsten inert gas (TIG) welding based on numerical simulation. Int. J. Adv. Manuf. Technol..

[23] Omprakasam S., Marimuthu K., Raghu R., Velmurugan T. (2022). Statistical modelling and optimization of TIG welding process parameters using Taguchi’s method. Stroj. Vestn.-J. Mech. Eng..

[24] Zhang D.K., Yue Z.H.A.O., Dong M.Y., Wang G.Q., Wu A.P., Shan J.G., Meng D.Y., Liu X.L., Song J.L., Zhang Z.P. (2019). Effects of weld penetration on tensile properties of 2219 aluminum alloy TIG-welded joints. Trans. Nonferrous Met. Soc. China.

[25] Gou W., Wang L. (2020). Effects of Welding Currents on Microstructure and Properties of 5052 Aluminum Alloy TIG Welded Joint//IOP Conference Series: Materials Science and Engineering. IOP Conf. Ser.: Mater. Sci. Eng..

[26] Ahmed S., Saha P. (2020). Selection of optimal process parameters and assessment of its effect in micro-friction stir welding of AA6061-T6 sheets. Int. J. Adv. Manuf. Technol..

[27] Vidyarthy R.S., Dwivedi D.K., Muthukumaran V. (2018). Optimization of A-TIG process parameters using response surface methodology. Mater. Manuf. Process..

[28] Zhou L., Luo L.Y., Wang R., Zhang J.B., Huang Y.X., Song X.G. (2018). Process parameter optimization in refill friction spot welding of 6061 aluminum alloys using response surface methodology. J. Mater. Eng. Perform..

[29] Chludzinski M., Dos Santos R.E., Churiaque C., Ortega-Iguña M., Sánchez-Amaya J.M. (2022). Effect of process parameters on pulsed laser welding of AA5083 alloy using response surface methodology and pulse shape variation. Int. J. Adv. Manuf. Technol..

[30] Salleh M.N.M., Ishak M., Quazi M.M., Aiman M.H. (2018). Microstructure, mechanical, and failure characteristics of laser-microwelded AZ31B Mg alloy optimized by response surface methodology. Int. J. Adv. Manuf. Technol..

[31] Tsirkas S.A. (2018). Numerical simulation of the laser welding process for the prediction of temperature distribution on welded aluminium aircraft components. Opt. Laser Technol..

[32] Balram Y., Rajyalakshmi G. (2019). Thermal fields and residual stresses analysis in TIG weldments of SS 316 and Monel 400 by numerical simulation and experimentation. Mater. Res. Express.

[33] (2012). Welding Procedure Qualification Test for Aluminum and Its Alloys.

[34] Wang Y., Cong B., Qi B., Yang M., Lin S. (2019). Process characteristics and properties of AA2219 aluminum alloy welded by double pulsed VPTIG welding. J. Mater. Process. Technol..

[35] Hakem M., Lebaili S., Mathieu S., Miroud D., Lebaili A., Cheniti B. (2019). Effect of microstructure and precipitation phenomena on the mechanical behavior of AA6061-T6 aluminum alloy weld. Int. J. Adv. Manuf. Technol..

[36] Ahmadi E., Ebrahimi A.R., Hoseinzadeh A. (2020). Microstructure Evolution and Mechanical Properties of 2219 Aluminum Alloy A–TIG Welds. Phys. Met. Metallogr..

[37] Qin Q., Zhao H., Li J., Zhang Y., Zhang B., Su X. (2020). Microstructures and mechanical properties of TIG welded Al-Mg2Si alloy joints. J. Manuf. Process..

[38] Baskoro A.S., Amat M.A., Pratama A.I., Kiswanto G., Winarto W. (2019). Effects of tungsten inert gas (TIG) welding parameters on macrostructure, microstructure, and mechanical properties of AA6063-T5 using the controlled intermittent wire feeding method. Int. J. Adv. Manuf. Technol..

[39] Samiuddin M., Li J.L., Taimoor M., Siddiqui M.N., Siddiqui S.U., Xiong J.T. (2021). Investigation on the process parameters of TIG-welded aluminum alloy through mechanical and microstructural characterization. Def. Technol..

[40] (2022). Safety Requirements for Bicycles—Part 2: Requirements for City and Trekking, Young Adult, Mountain and Racing Bicycles.

